# Neurocognitive sparing of desktop microbeam irradiation

**DOI:** 10.1186/s13014-017-0864-2

**Published:** 2017-08-11

**Authors:** Soha Bazyar, Christina R. Inscoe, Thad Benefield, Lei Zhang, Jianping Lu, Otto Zhou, Yueh Z. Lee

**Affiliations:** 1 Department of Biomedical Engineering, The University of North Carolina at Chapel Hill, 350 Chapman Hall, 4Chapel Hill, NC 27599 USA; 2 Department of Applied Physics Sciences, The University of North Carolina at Chapel Hill, Chapel Hill, USA; 3 Department of Physics and Astronomy, The University of North Carolina at Chapel Hill, Chapel Hill, USA; 4 Biomedical Research Imaging Center, The University of North Carolina at Chapel Hill, Chapel Hill, USA; 5 Lineberger Comprehensive Cancer Center, The University of North Carolina at Chapel Hill, Chapel Hill, USA; 6 Department of Radiology, The University of North Carolina at Chapel Hill, CB#7510, Chapel Hill, NC 27599 USA

**Keywords:** Microbeam radiation therapy, Radiation-induced cognitive Impairment, Brain radiotherapy, Spatially fractionated radiotherapy, Carbon nanotube x-ray

## Abstract

**Background:**

Normal tissue toxicity is the dose-limiting side effect of radiotherapy. Spatial fractionation irradiation techniques, like microbeam radiotherapy (MRT), have shown promising results in sparing the normal brain tissue. Most MRT studies have been conducted at synchrotron facilities. With the aim to make this promising treatment more available, we have built the first desktop image-guided MRT device based on carbon nanotube x-ray technology. In the current study, our purpose was to evaluate the effects of MRT on the rodent normal brain tissue using our device and compare it with the effect of the integrated equivalent homogenous dose.

**Methods:**

Twenty-four, 8-week-old male C57BL/6 J mice were randomly assigned to three groups: MRT, broad-beam (BB) and sham. The hippocampal region was irradiated with two parallel microbeams in the MRT group (beam width = 300 μm, center-to-center = 900 μm, 160 kVp). The BB group received the equivalent integral dose in the same area of their brain. Rotarod, marble burying and open-field activity tests were done pre- and every month post-irradiation up until 8 months to evaluate the cognitive changes and potential irradiation side effects on normal brain tissue. The open-field activity test was substituted by Barnes maze test at 8th month. A multilevel model, random coefficients approach was used to evaluate the longitudinal and temporal differences among treatment groups.

**Results:**

We found significant differences between BB group as compared to the microbeam-treated and sham mice in the number of buried marble and duration of the locomotion around the open-field arena than shams. Barnes maze revealed that BB mice had a lower capacity for spatial learning than MRT and shams. Mice in the BB group tend to gain weight at the slower pace than shams. No meaningful differences were found between MRT and sham up until 8-month follow-up using our measurements.

**Conclusions:**

Applying MRT with our newly developed prototype compact CNT-based image-guided MRT system utilizing the current irradiation protocol can better preserve the integrity of normal brain tissue. Consequently, it enables applying higher irradiation dose that promises better tumor control. Further studies are required to evaluate the full extent effects of this novel modality.

**Electronic supplementary material:**

The online version of this article (doi:10.1186/s13014-017-0864-2) contains supplementary material, which is available to authorized users.

## Background

Annually, approximately 200,000 new cases of malignant brain tumors are diagnosed in the US alone [1, 2]. Radiotherapy (RT) has remained an important treatment modality for intracranial tumors despite the inevitable normal tissue toxicity, which is the primary reason for dose limitations. As treatment modalities have improved, patients live long enough to experience radiation-induced brain injury [3, 4]. Accordingly, the American Cancer Society has stressed that future research should focus on reducing the complications of radiotherapy to maximize the quality of life for patients after treatment [4]. Microbeam radiation therapy (MRT) is a promising pre-clinical approach in spatially fractionated RT, which delivers quasi-parallel submillimeter lines of high-dose irradiation (beams) that are separated by wider non-irradiated regions (valleys). The majority of MRT studies have been limited to synchrotron facilities. However, aside from the sparseness of these facilities, the lack of specialized hospitals near them has also severely hindered the translation of this promising treatment approach to millions of patients around the world.

To make this technology more available for preclinical biomedical studies, we have developed the first desktop MRT device based on the spatially distributed carbon nanotube x-ray technology (CNT) [5], which enables delivering a high dose of radiation in a laboratory setting. Our system uses multiple concurrently activated cold cathodes sources arranged in a line. By distributing the electron beam along a very long and narrow line on the anode instead of a single point, significantly better heat conduction and therefore, higher dose delivery rates can be achieved as compared to conventional point-focused X-ray tubes. Furthermore, the radiation can be readily gated with physiological signals during irradiation [6].

Nowadays, using current radiation approaches, acute (days to weeks after irradiation) and subacute (1–6 months post-irradiation) radiation-induced brain injuries are rare and reversible, while the delayed injuries (6 months to 1-year post-irradiation) are irreversible and progressive [7]. In addition, the volume of normal brain that is irradiated (the field size) is an important toxicity determinant. Most of the studies on the effect of MRT on normal brain tissue are focused on the short time outcome after whole- or one-hemisphere-brain MRT [8–10]. Consequently, more recently, many groups, including Smyth et al. [11], have emphasized the importance of evaluating chronic irradiation-induced changes by MRT treatment on a confined area of the brain. In the previous studies, we found that applying image-guided MRT using our novel method was able to induce tumor control in intracranial murine tumor model, without causing any significant histological changes up to 30 days post-irradiation [12, 13]. However, our histology evaluations indicated that BB might cause more normal brain tissue damage than MRT in later time-points [13]. Consequently, we hypothesized that applying image-guided MRT using our novel method would elicit less neurocognitive impairment than equivalent BB irradiation in long-term follow-up. Here, our goal was to evaluate the potential effects of MRT on normal brain tissue and compare it with conventional broad beam (BB) post-irradiation in acute, subacute and more importantly, the chronic time intervals.

## Methods

### Animals

Four-weeks-old male C57BL/6 J mice (Jackson Laboratory, Bar Harbor, ME) were acquired and allowed to acclimate for a week before study initiation.

The mice were housed in the University of North Carolina at Chapel Hill (UNC-CH) Division of Laboratory Animal Medicine (DLAM) pathogen free designated environment and cared for in accordance with the United States Department of Health and Human Services Guide for the Care and Use of Laboratory Animals; all procedures were approved by UNC-CH Institutional Animal Care and Use Committee (IACUC). Mice were housed in a temperature and light-controlled environment with 12-h light/dark cycle (lights on at 7 AM) and provided food and water.

### Irradiation

Mice were randomly assigned to three treatment groups: microbeam radiotherapy, broad-beam radiotherapy and sham. All the mice underwent treatment at eight weeks old under anesthesia with 1–2.5% isoflurane in medical-grade oxygen at 0.8–1 L/min flow rate. All mice kept anesthetized for an equal duration of time (two hours) to normalize the influence of isoflurane on behavioral tests outcomes [14–16].

### Dosimetry

GAFCHROMIC™ EBT3 (Ashland Advanced Materials, Covington, KY, US) film was placed at the dose entrance plane for dosimetry and evaluating the dose profiles. The key technical features of GAFCHROMIC™ EBT3 films that make them suitable for our purpose included the minimal response difference over a wide photon energy range and high spatial resolution (25 μm or higher) [17]. As a result, several MRT studies have used these radiochromic films for the dosimetry evaluations [5, 18–20].

The film was cross-calibrated to an ion chamber and scanned as previously described [21, 22]. Scanned films were processed using in-house written Matlab script (R-2015a, The MathWorks, Inc., Natick, MA) using principles described by Borca et al. [23].

### Microbeam radiotherapy

**Fig. 1:**
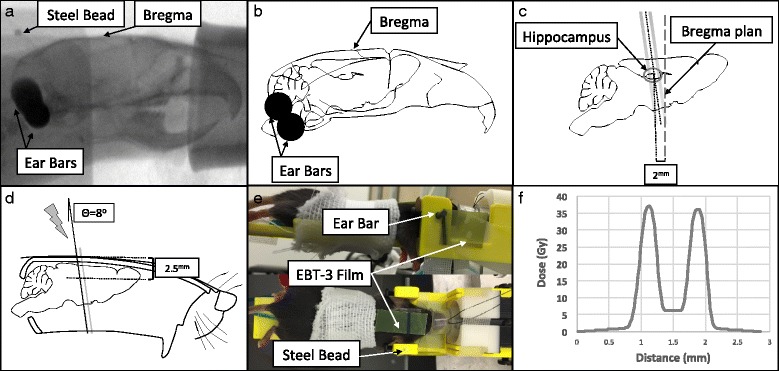
Image-guided Microbeam Radiotherapy Method Abstract. **a:** Lateral radiograph of mouse head was taken to locate the bregma. The head was stabilized using two ear-bars and teeth wire. Embed steel bead served as the fiducial marker. **b:** The skull outlines were sketched over the same radiograph. The anatomical place of hippocampus is shown regarding the bregma. **d:** Schematic lateral view of mouse skull with a cut along the corpus callosum at midline. The *gray line* demonstrates the microbeam. In our device, the microbeam planes intersect with the vertical plane at a slight angle of 8 degrees. The center of the treatment was placed 2 mm posterior to the bregma (**c**) and 2.5 mm inferior to the top of the skull [50]. **e:** The side (*top*) and top view (*bottom*) of mouse under irradiation. The head was fixed by ear bars and tooth wire. Gafromic EBT-3 film was placed on top of the mouse head (entrance plan) to record the beams and generate the dose profile (**f**)

Image-guided MRT was performed on normal mice brains by desktop CNT-based MRT system. Image-guided radiotherapy was conducted using the protocol as reported previously [21]. In brief, lateral X-ray projections were taken using onboard micro-CT scanner to locate the bregma (Fig. 1a,b). An embedded steel bead (1/32 in. ≈ 0.8 mm) in the holder was used as the fiducial landmark (Fig. 1a). Since the microbeam planes intersect with the vertical plane at a slight angle of 8 degrees (collimator angle) [22], it was crucial to calculate the distance to the center of hippocampus from the registered images in both anterior-posterior and superior-inferior directions (Fig. 1c,d).

**Fig. 2:**
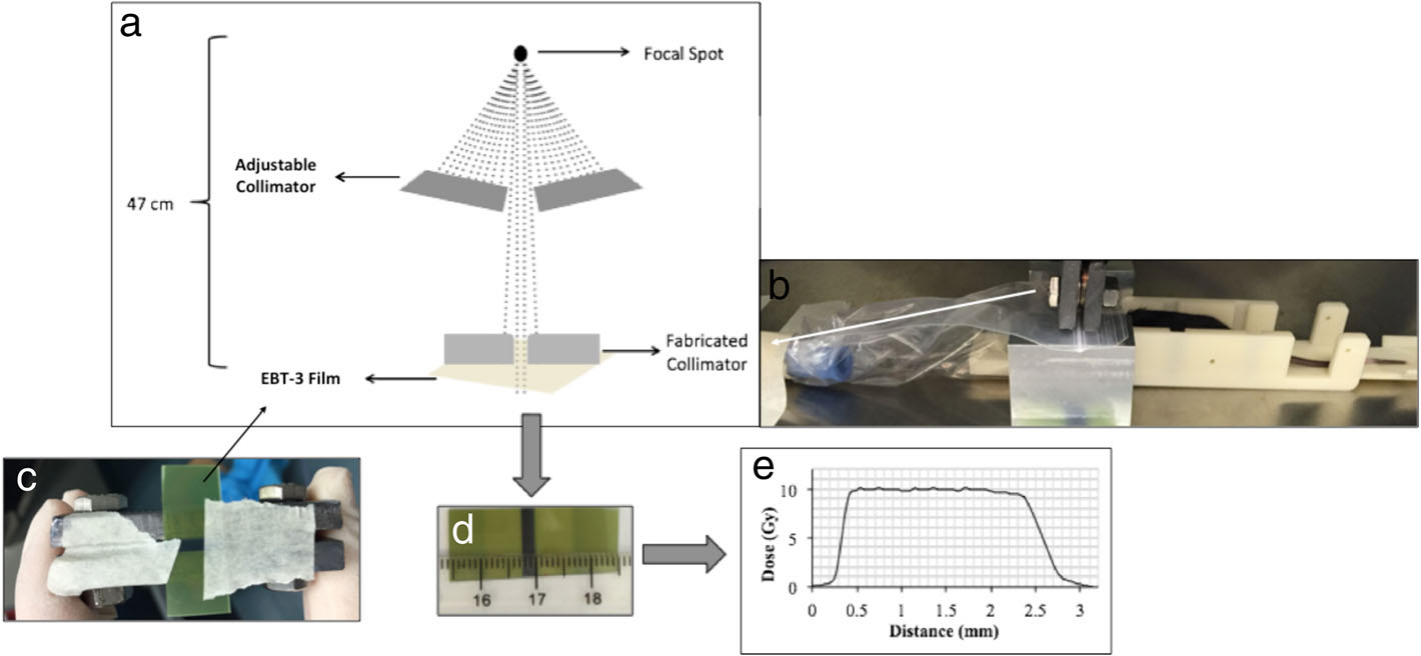
Broad-beam (BB) Irradiation Method Abstract. **a**: The schematic picture demonstrates the steps to collimate down the beam to 2.5 mm (**d**). **b**: The fabricated collimator was placed in close contact with mouse head. **c:** The Gafchromic™ EBT-3 film attached to fabricated collimator to record the entrance dose (**d**) and generate the beam profile (**e**)

After imaging, the mice were mechanically translated from the imaging to the irradiation position. Detailed descriptions of the device and dosimetry have been previously reported [22]. Two arrays of microbeams were delivered unidirectional along the coronal plane across each mouse brain (Fig. 2e). Each microbeam was 300 μm wide, spaced at 900 μm center-to-center distance and the radiation field was centered on the hippocampus (2 mm posterior and 2.5 mm inferior to bregma) (Fig. 1c,d). The peak dose was 36 Gy and 5 Gy dose of X-ray was manually deposited in valley area (Fig. 1f).

### Broad-beam radiotherapy

**Fig. 3:**
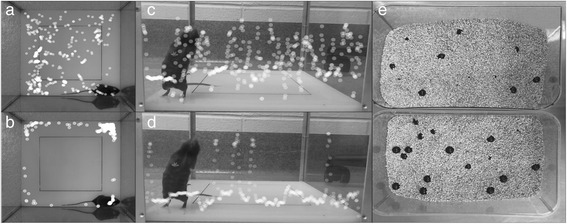
Comparison of Normal Mouse (*top row*) vs. Impaired One (*bottom row*). The white dots are the position of mouse neck (junction of head and body) at each second during first 10 mins of open-field activity test (superimposed scatter plots are generated using idTracker [62]). The impaired mouse spends more time at periphery (**a** vs. **b**) and did less rearing (**c** vs. **d**) and buried fewer marbles after 30 min test (**e** *top* vs. *bottom*)

An industrial X-ray machine (X-RAD 320, PXi, North Branford, CT) was used for the BB irradiation. The dose rate, after 1.5 mm aluminum, 0.25 mm copper, plus 0.75 mm tin filter, was 1.06 Gy/min at a focal surface distance of 47 cm (Fig. 2a). For BB irradiation, the hippocampal area was irradiated with 10 Gy of X-ray over 2.5 mm irradiation field, creating an integrated equivalent dose to the MRT beams. The beam was collimated down to 10 mm wide using an industrial 4-leaf adjustable collimator (PXi, North Branford, CT) and then further collimated to 2.5 mm using fabricated collimator out of 1.5 cm plates of lead (Fig. 2a,b). The setting applicability was pretested and the dose was measured using GAFCHROMIC™ EBT3 film (Fig. 2c,d). During the experiment, the mice were positioned such that their heads were in close contact with the fabricated collimator and stabilized using ear bars and nose cone (Fig. 2b). The collimator was placed 1 mm anterior to the interaural line to target hippocampus. The orientation of beam was same as MRT (Fig. 3c-e).

### Neurocognitive testing

Mice were assessed using a battery of neurocognitive function tests at baseline and at each month after treatment and weighed using a scale with the accuracy of 10^−1^ g every week for the period of study. To minimize the effects of social influences on the behavior, mice were housed three in a cage, in which there was one member of each group. All the experiments were performed between 9:00 am-3:00 pm during consecutive days of a week. The baseline weight and measurements of rotarod, open-field and marble burying were compared between treatment groups, to make sure no baseline difference existed among treatment groups. A pretest was performed during which mice were evaluated pre-treatment and every week up until one month and every month post-irradiation up until three months to evaluate the appropriate time point to perform the behavioral test Additional file 1: Figure S1.

### Rotarod

Mice were placed on a cylinder, which slowly accelerates to a constant rotating speed. While the heads of the mice are placed against the direction of the rotating rods, normal mice learn to walk forward as the rod rotating-speed increases. For each trial, revolutions per minute (rpm) were set at an initial value of 3 and progressively increased to a maximum of 30 rpm across 5 min. In all test sessions, the time latency before the mouse lost its balance was measured in seconds, up to maximum 300 s.

### Pre-treatment training

An accelerating rotarod (Acceler. Rota-rod (Jones & Robertson) for mice, 7650, Ugo Basile, Varese, Italy) was used for the acquisition of the task. For the first session, mice were given 3 trials, with 45 s between each trial. A second test session with 2 trials was conducted 48 h later, to evaluate consolidation of motor learning.

### Post-treatment evaluation

A similar accelerating rotarod was used for the re-evaluation of motor coordination. For each test, mice were given 2 trials, with 45 s between each trial.

### Open-Field activity

Novel environment exploration, general locomotors activity, and anxiety-related behaviors in rodents were assessed systematically within a square 41 cm × 41 cm Plexiglas® box. Mice were filmed during the 30 min trial. Measures were taken of the number of the rearing (frequency with which the mice stood on their hind legs) and duration of time they spend doing locomotion and in the central square (29 cm × 29 cm, 50% of field area) vs. periphery in both baseline and post-treatment assessments.

A high duration of locomotion behavior and time spent in the central square indicate increased exploration and a lower level of anxiety [24]. It had been shown that anxiolytics administration increases exploration time in the center of the open-field while stressful stimuli decrease the number of center visits [24] (Fig. 3a-b). Open-field activity, therefore, represents a valid measure of marked changes in “anxiety-like” behaviors [25]. In addition, rearing frequency corresponds with hippocampal electrical activity [26] (Fig. 3c-d).

### Pre-treatment

Mice were assessed by 30 min trial in an open-field arena, crossed by a grid of photobeams. Counts were taken of the number of photobeams broken during the half an hour trial either horizontally or vertically (VersaMax, AccuScan Instruments).

### Post-treatment

Mouse activity was recorded during 30 min experiment in the same size arena and assessed for the same parameters using different software (The Observer XT 10, Noldus Bv, Wageningen, The Netherlands).

### Marble burying

Digging is a species-specific behavior of mice. It has been shown that hippocampal lesions markedly reduces the number of buried marbles to the point that cages of mice with hippocampal lesions appears to have had no mice in them at all [27] (Fig. 3d). To quantify this behavior, twenty 9/16″ (14.3 mm) black glass marbles were placed in equally distance five row and four columns in a 28 × 17 × 10 cm clear plastic cage, two third of which was filled with bedding. The cages were covered thoroughly after putting the mice in them. The number of buried marbles was counted after 30 min. Buried marble was defined as the one that more than half of it was in the bedding.

### Barnes maze

During the test, a mouse was placed at the center of a 92 cm circular table around which there were 20 holes each 5 cm along the edges. Animals escaped from a brightly lit open arena into a small basket located under one of the openings. The opening to place the basket under was assigned for each mouse randomly and remained the same all along the testing period. The Barnes Maze platform was made in-house using measurements from Sunyer et al. [28]. Printed patterned papers were placed in different places in the room as spatial cues. Mice were tested for 7 consecutive days and measure was the duration of time before finding the right opening. Each test session was up until they enter the escape box or up to 5 min. If mice were not able to find the correct opening during the test the period, they were gently directed toward it.

The mice were evaluated by the Barnes maze test 8-month post-irradiation (to measure chronic effect). At this time point, the open-field activity was not performed because both tests are based on the fear of isolation and being exposed in brightly lit areas, and one test may have a negative effect on the other one’s results.

### Immunohistochemistry

Brain tissues from the animal were collected at the end of the 3rd- (pretest group) and 9th-month post treatment. Whole mouse brains were fixed in formalin for 48 h, processed, embedded in paraffin, serially sectioned at 5 μm thickness and were used for IHC.

IHC was carried in Bond the fully automated immunostainer (Leica). Slides were dewaxed in Bond Dewax solution (AR9222) and hydrated in Bond Wash solution (AR9590). Hematoxylin and Eosin (H&E) stain was done in the Autostainer XL (Leica Biosystems Inc., Vista, CA). H&E stained slides were digitally imaged in the Aperio ScanScope XT (Leica) using 20× objective. The complete list of the stains used to evaluate the histological changes 3-month post-irradiation can be found in the Additional file 2.

### Statistical analysis

Statistical analysis was performed by SAS/STAT^®^ version 9.4 (SAS Institute Inc., Cary, North Carolina). A *p*-value <0.05 was considered statistically significant. The means of baseline values were compared using ANOVA to ensure there was no significance difference at baseline among treatment groups. A multilevel model, random coefficients approach was used to make inferences concerning treatment group differences. Random coefficient models allow simultaneous inferences at the aggregate and individual level while accounting for correlation between subjects that arises in longitudinal studies. These models are also more flexible than traditional ANOVA approaches because the constraint that each subject has the same regression coefficients is removed. Random coefficient models are also more powerful than standard cross-sectional methods with appropriate multiple comparison controls. For each outcome, the level 1 regression equation was found using the partial residual sum of square (PRESS) statistic under 5-fold cross validation to determine the order of the polynomial fit.

Fitting the polynomial structure discovered using the above method; we chose the order of the random effects that would minimize BCC in the unconditional models while yielding nonzero covariance for the highest order term. Each random coefficient was modeled as a function of treatment group, engendering the level 2 regression equations. Interactions with treatment group and time arising from the level 2 equations were assessed using type 3 tests and dropped where they were not significant. When the treatment group was found to predict linear or higher order slope terms, regions of significance were calculated. Tests of differences in treatment groups were conducted where the treatment group was found to predict intercepts only.

## Results

**Fig. 4:**
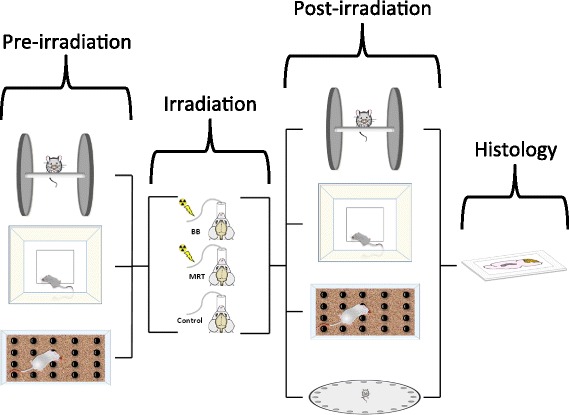
Method Abstract. The mice were pre-evaluated using rotarod, open-field activity and marble burying tests were randomly assigned to three treatment groups: broad beam (BB), microbeam radiotherapy (MRT) and controls. All mice were maintained under gaseous anesthesia for the equal duration of time. The post-irradiation evaluations were performed each month by rotarod, open-field activity and marble burying and 8-month after exposure Barnes maze test was used to evaluate the mice. All mice brains were sent for histological assessments 9-month post-irradiation

Figure 4 demonstrates a schematic flowchart of current study. Mice were weighted and pre-evaluated using a series of cognitive tests and randomly assigned in three treatment groups (see pre-irradiation Fig. 4). No significant differences among MRT, BB and shams in any of measurements at baseline (Table 1).

**Table 1:** Pre-irradiation Evaluation of the Mice in Three Groups

Measurement		*P*-value
Weight		0.576
Rotarod		0.365
Marble Burying		0.216
Open-field Activity	Rearing	0.332
	Center	0.506
	Locomotion	0.241

Mice brains in MRT and BB groups were irradiated with integrated equivalent dose (irradiation phase Fig. 4). All mice in MRT and BB groups tolerated the irradiation procedures well, with no specific veterinary concerns. Acute skin effects (erythema, desquamation, inflammation or epilation) were not detected in any mice after any irradiation approach.

Histological studies [13] and pretest results (Additional file 1: Figure S1) demonstrated no measurable changes during the acute phase post-irradiation (up until one month) and as a result, the mice were evaluated every month post-irradiation using a battery of test in the current study as demonstrated in Fig. 4, post-irradiation phase.

**Fig. 5:**
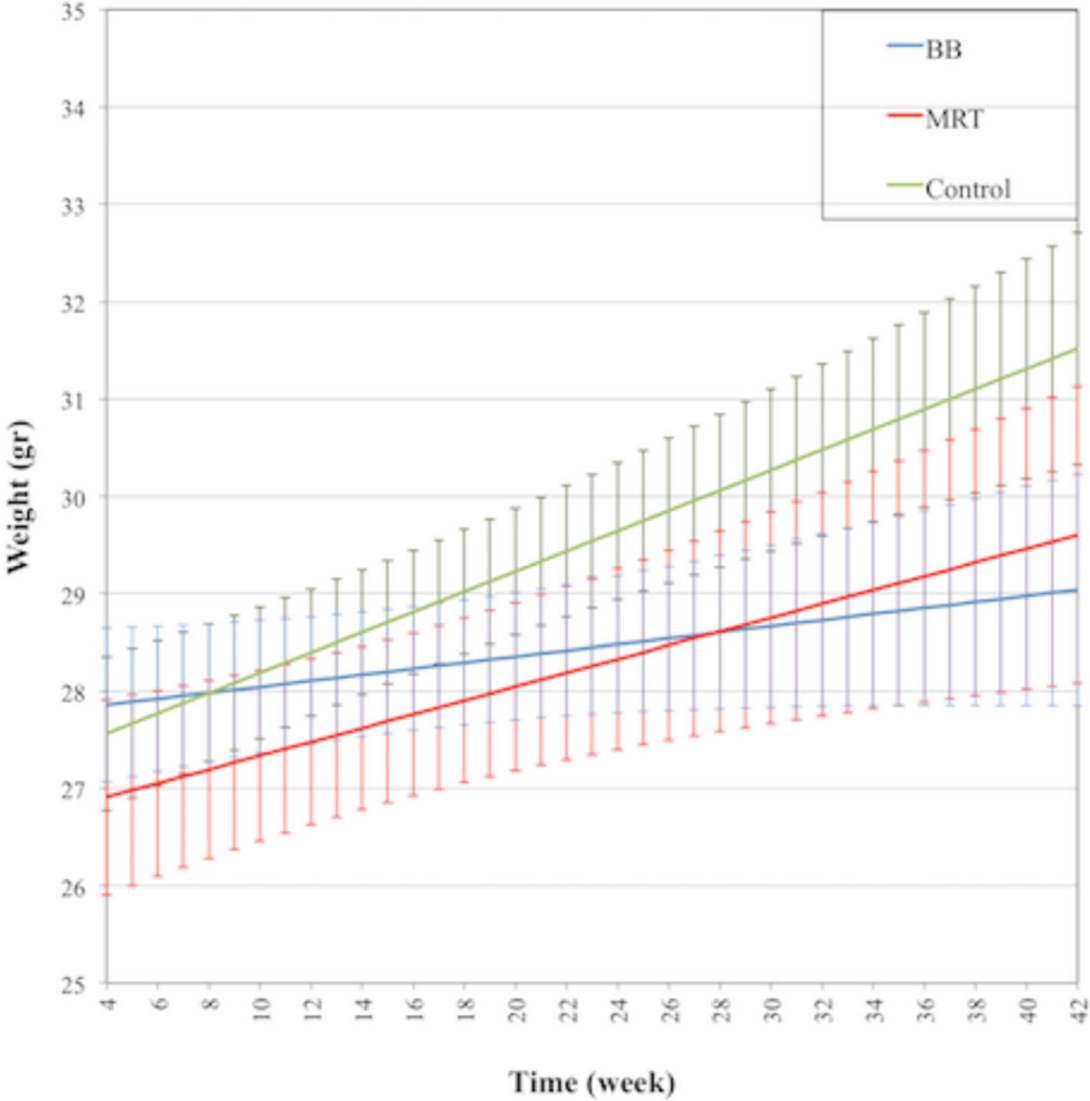
Predicted Mean of Mice Weight. The mice were weighed at their arrival to the facility and each week after irradiation. The error bars are SD

The BB mice, whose brains were irradiated with homogeneous 10Gy of X-ray using a 2.5 mm wide beam, tended to gain weight at a slower rate than MRT and non-irradiated mice. This difference became statistically significant between BB and controls since week 31 post-irradiation until the end of the experiment (week = 42) (Fig. 5).

There was no difference in the duration of time mice kept their balance on the rotating rod, duration of rearing and duration of time mice spent in the central area of the open-field arena by treatment group (Table 2).

**Table 2:** Post-irradiation Longitudinal Neurocognitive Evaluation

Behavioral Tests		Group Difference ^a^	*P*-value
Rotarod		MRT vs. Control vs. BB	0.520
Marble Burying		MRT-Control = 0.0572	0.910
		MRT-BB = 1.410	0.011^+^
		Control-BB = 1.353	0.009^+^
Open-field Activity	Rearing	MRT vs. Control vs. BB	0.180
	Center	MRT vs. Control vs. BB	0.510
	Locomotion	MRT-Control = 35.211	0.235
		MRT-BB = 120.50	0.0005^+^
		Control-BB = 85.291	0.005^+^
Barnes Maze		MRT-Control = 3.549	0.861
		MRT-BB = −36.298	0.085
		Control-BB = −39.847	0.044^+^

^+^ *P*-Value < 0.05

^a^ The difference between predicted means are reported when there was statistically significant difference among groups

The number of the buried marbles in BB was significantly less than the control group and significantly less than the MRT group at all time points for (*p* ≤ 0.01) and BB mice spent less time searching around the open-field arena (*p* < 0.001).

BB mice spent more time finding the right hole in the Barnes maze test than shams in all test sessions (*p* = 0.044). There were no differences between the MRT and shams for either of these outcomes.

Interestingly, at five-month post-irradiation, a depigmented line appeared in all BB mice at the site of irradiation (Additional file 3: Figure S2) which progressed until 6th month and remained the same without any regression or progression for the duration of the study (up to 9-month post-irradiation). In two out of eight mice in MRT group, a line of gray hair appeared in the exit plan at the beginning of 8-month after irradiation that stopped progression after 20 days and did not regress during next two months.

The brain tissues of the mice were collected 4 and 9 months post-treatment in pretest and test studies, respectively (Fig. 4 histology). No histological changes were detected in any mice brain sample using IHC (data not shown).

## Discussion

Radiation-induced cognitive impairment is the most frequent complication among long-term cancer survivors and occurs in up to 50–90% of adult brain tumor patients who survive more than 6 months post fractionated partial or whole brain irradiation [29–32]. In spite of adequate disease control, cognitive impairment interferes with the patients’ ability to function at their pretreatment levels. Multiple prior animal studies have reported that synchrotron MRT induces less neurotoxicity than conventional radiotherapy [33, 34]. Here, we found that MRT using first generation CNT-based image-guided desktop microbeam irradiator would also cause less neurocognitive impairment than equivalent BB irradiation. To the best of our knowledge, this is the first time that MRT and BB radiation-induced cognitive impairments have been investigated using such a comprehensive battery of behavioral assessments for a long duration of time after irradiation.

Local irradiation of hippocampal area with 10 Gy led to declined cognitive function in BB mice compared to sham (See Table 2). It has been found that 8-month after X-ray irradiation of mouse brain with 10 Gy, there was significant inhibition in neurogenesis level at hippocampus [35]. These may explain the decline in BB mice cognitive level in the current study at 8th-month post-irradiation measured using Barnes maze test.

Interestingly, no significant difference was found between MRT and shams at any time points post-irradiation. Different studies have reported that brain normal tissue can maintain its normal function and integrity at higher doses of X-ray in MRT than conventional radiotherapy methods. Four main mechanisms have been postulated to play crucial role in keeping the normal tissue integrity after MRT. First, a “beneficial” bystander effect is hypothesized to facilitate the restoration of injured cells in central nervous system [36]. Second, due to the unique spatial distribution of X-ray in MRT, the total contact surface between highly irradiated and damaged tissue along the beam and minimally irradiated valley area is increased which may allow cells in the valley to maintain the function of the normal tissue. Third, multiple studies revealed that normal brain macro and microvasculature shows higher tolerance to MRT and immature vessels like tumor neovasculature are preferentially damaged by this method [37]. At last, recently, it has been shown that a spectrum of immune response would be evoked. While part of this response is in favor of normal tissue damage, different immune responses are evoked in favor of tumor resolution and preserve the normal tissue function [38, 39]. Interestingly, studies have demonstrated that activated immune responses after MRT favor this latter effect [40, 41].

No acute skin effects were observed in any mouse after broad- or micro-beam radiotherapy. In the current study, we observed the depigmentation hair circle in all BB-treated mice at the site of irradiation (Additional file 1: Figure S2). Kinoshita et al. also observed the same effects when locally irradiated C57BL/J6 mice by a single fraction of 10 Gy [42]. Microbeam radiation therapy utilizes relatively low beam energies to keep the spatial fractionation deep in the tissue (an anode voltage energy of 160 kVp was used in the present study), which results in the lower dose penetration than the conventional radiotherapy. As a consequence, a significantly higher dose to the skin’s surface needs to be applied during MRT to ensure an adequate dose delivery to the target tissue. Paradoxically, in multiple microbeam therapy studies higher than normal tolerance of normal skin tissue has been observed [11, 43]. Interestingly, a line of gray hair appeared in two mice in MRT group at the exit plan 8-month after X-ray exposure (See Additional file 3). Previous studies have shown that skin effects are more severe at the joint places like axilla, groin and toes where the skin is subject to friction, or has folds in its surface [44]. Since this line coincides with the junction of mouse head and neck, we hypothesized that this effect may be due to the constant motion of these tissues with the associated inflammation.

No significant histological differences were detected 4- and 9-month post-irradiation based on light microscopy level (data not shown). While some hypothesized that neurocognitive changes may precede histological changes, a growing number of studies have correlated the radiation-induced cognitive deterioration to changes in the subcellular and molecular level of neuronal function and plasticity, particularly hippocampal long-term potentiation (LTP) [45]. These changes can happen even after a modest dose of X-ray (2–10 Gy) [46].

It is well established that the hippocampus plays a crucial role in learning and memory and its damage leads to various behavioral alterations including spatial learning impairment and disturbances in fear/anxiety responses [47, 48]. Given these critical roles and the importance of hippocampal sparing radiotherapy in clinical applications [49], we focused on the hippocampus as the target of our treatment and used a radiation field size to cover the whole mice hippocampus [50]. As a consequence, our chosen behavioral tests were focused to evaluated hippocampal-associated function (see Neurocognitive Testing under Method).

Here, we mimicked clinical irradiation protocols, so we applied a local low X-ray dose that we knew would induce cognitive impairments [51], but was well below the threshold for inducing obvious histological changes. Due to the distinct spatial fractionation of X-ray beam in MRT, finding the actual equivalence dose of MRT is convoluted. Previous studies have used different assumption for the physical or biological equivalent dose [41, 52]. Priyadarshika et al. suggested that the integrated dose of MRT, which is the microbeam dose averaged over the entire radiation volume, might be more relevant than the peak or valley dose when compared to broad-beam radiation [53]. In previous study, we found that 10Gy of the BB would induce same treatment efficacy as the integrated MRT dose [13]. Accordingly, here we also assumed that integral dose is close to actual equivalent dose, so for MRT group an identical anatomical region of the brain was irradiated with the equal integrated dose.

The peak-to-valley dose ratio (PVDR) has been measured 16 at the entrance plane and decreased to 14 at the exit plane, so the equivalent integral dose of 10Gy BB simulated to be ≈ 46Gy in peaks [13]. But several histological studies after high dose brain MRT have shown a discrete band of neuronal and glial nuclei loss only along the beam path [54–57]. This observation supports the idea that surviving cells in the valley region play the main role in maintaining tissue function and compensating for the loss of functional cells in the peak region. Consequently, after microbeam irradiation, brain toxicity is more dependent on valley region parameters [11]. The average dose rate at the mouse brain entrance plane has been measured to be 1.2 Gy/min. As a result, to keep the total duration of the procedure under 2 h, according to IACUC approved protocol, we selected a peak X-ray dose of 35 Gy with a valley dose 5 Gy, to increase the toxic effect of our method.

Our study has following limitations. The total number of mice was limited (*n* = 24 in the test), but by running pretest (*n* = 9 in pretest), and use of different tests on separate days, we had increased sensitivity to detecting subtle differences. On 8th-month post-irradiation, the mice were evaluated using Barnes Maze test, which has been found to be the most sensitive test for detection of irradiation-induced hippocampal-dependent cognitive changes in rodent [51]. Another limitation was the use of normal mice. Patients with brain tumors often experience cognitive dysfunction associated with the disease that is present at diagnosis [39, 58]. As a result, tumor regression will substantially improve the neuropsychological function level [59]. In the current study, the effect of two different methods of radiotherapy on normal healthy mouse brain was compared. Having said that, a recent study has shown that brain tumor patients are more prone to post-irradiation cognitive deterioration than normal patients [60]. Consequently, the optimal study would be the one that compares the neurocognitive of BB- and MRT-treated brain tumor mice. However, considering the aggressive nature of mice brain tumor models, such study is not feasible for a long time follow-up.

## Conclusions

We found that microbeam radiotherapy using our desktop device and the irradiation protocol we utilized in the current study induced less neurocognitive impairment than the same integrated uniform dose on the hippocampal area in normal mice up to 8-month post-irradiation. Our previous studies demonstrated that applying MRT using our device is able to control the murine model of glioblastoma effectively [12]. This suggests that another potential advantage of MRT in brain tumor treatment is improved local tumor control rates with the ability to apply radiobiological higher doses either by re-irradiating of the same lesion using the same method or combining other radiation modalities. Brain tumors are the most common solid tumor in pediatrics [61] and MRT seems to be a promising treatment modality for this group of patient. Thus, in the future study, we aim to evaluate the effect of this treatment on immature rodent brain.

## Additional files

Additional file 1: Pretest results. (DOCX 754 kb)

Additional file 2: Supplementary Method. (DOCX 16 kb)

Additional file 3: Picture of a BB-treated mouse head 6 months post-irradiation. The arrow points to the circle of gray hair at irradiation site. (DOCX 1533 kb)

## Abbreviations

BB: Homogenous broad beam irradiation;
CNT: Carbon nanotube X-ray technology
DLAM: Division of Laboratory Animal Medicine
IACUC: Institutional Animal Care and Use Committee
IHC: Immunohistochemistry
LTP: Long-term potentiation
MRT: Microbeam irradiation therapy
PRESS: Partial residual sum of square
RT: Radiotherapy
UNC-CH: University of North Carolina at Chapel Hill
