# Supplementary material for: Neurocognitive sparing of desktop microbeam irradiation
Source: Radiat Oncol. 2017 Aug 11;12:127. doi: 10.1186/s13014-017-0864-2 (PMC5554005; doi:10.1186/s13014-017-0864-2)
Supplement: Supplementary file 1 — Pretest results. (DOCX 754 kb) [file 13014_2017_864_MOESM1_ESM.docx]

Additional file 1: **Figure S1 Pretest results.** The graphs show the mice (right column) measured every day and mean of the number of rearing and duration of time mice in each group spent in the central (middle column) during 600 sec of open-field activity test in pretest mice (9 mice). The right column demonstrates the mean time mice spent on the rotarod (300sec/trails, 2 trials), and means the number of marbles they buried after 30min. Statically significant differences are highlighted. The duration of time mouse spent in locomotion around the activity arena was not different at any time point post irradiation (data not shown here). The data are shown as mean±SD.
