# Supplementary material for: Neurocognitive sparing of desktop microbeam irradiation
Source: Radiat Oncol. 2017 Aug 11;12:127. doi: 10.1186/s13014-017-0864-2 (PMC5554005; doi:10.1186/s13014-017-0864-2)
Supplement: Supplementary file 2 — Supplementary Method. (DOCX 16 kb) [file 13014_2017_864_MOESM2_ESM.docx]

**Additional file 2: Supplementary Method**

**Pretest Immunohistochemistry:**

For pretest mouse, In addition to H&E, following rabbit polyclonal antibodies were used: cleaved caspase-3 (cC3), #CP229C (Biocare Medical, Concord, CA), CD11b, #NB110-89474 (Novus Biologicals, Littleton, CO), CD31, #ab28364 (Abcam, Cambridge, MA), GFAP, #Z0334 (Dako, Carpinteria, CA), Ki-67 #NCL-Ki67p (Leica Biosystems Inc., Vista, CA); Rabbit monoclonal anti-PCNA, clone EPR3821, #ab92552 was from Abcam (Cambridge, MA) and Rat monoclonal antibodies against F4/80 (#14-4801-82, clone bM8) and Myelin Basic Protein (MBP, #ab7349, clone 12) were from eBioscience (San Diego, CA) and Abcam (Cambridge, MA) respectively.

IHC and immunofluorescence were carried in Bond the fully automated immunostainer (Leica). Slides were dewaxed in Bond Dewax solution (AR9222) and hydrated in Bond Wash solution (AR9590). Antigen retrieval for all antibodies except cC3 was performed for 30 min at 100ºC in Bond-Epitope Retrieval solution1 pH-6.0 (AR9961) and in solution2 (pH9.0) for cC3.

After pretreatment slides were incubated for 30 min with PCNA (1:1000), CD11b (1:1500), GFAP (1:2500) and ki-67 (1:300) and for 60 min with cC3 (1:50), CD31 (1:200), F4/80 (1:50) and MBP (1:200). Detection of CD31, cC3, Ki-67, GDAP, CD11b and PCNA was performed using Bond™ Polymer Refine Detection system (DS9800). Detection of F4/80 was done using Bond Intense R Detection system (DS9263), supplemented with 1:500 Goat Anti-Rat (Biotin) secondary antibody (ab7096). Stained slides were dehydrated and coverslipped.

Immunofluorescent (IF) detection of MBP was done using Bond Intense R Detection kit (DS9263) supplemented with biotinylated Rabbit Anti-Rat Immunoglobulins (DAKO, E0468) and TSA-Cy3 (SAT704A001EA, Perkin Elmer) reagent; slides were counter-stained with Hoechst 33258 (Invitrogen) and mounted with ProLong Gold antifade reagent (P36934, Life Technologies). Positive and negative controls (no primary antibody) were included for each antibody. High-resolution acquisition (20x objective) of the IF slides (MBP) in the DAPI and Cy3 channels were performed in the Aperio ScanScope FL (Leica).
