# Supplementary material for: Neurocognitive sparing of desktop microbeam irradiation
Source: Radiat Oncol. 2017 Aug 11;12:127. doi: 10.1186/s13014-017-0864-2 (PMC5554005; doi:10.1186/s13014-017-0864-2)
Supplement: Supplementary file 3 — Picture of a BB-treated mouse head 6 months post-irradiation. The arrow points to the circle of gray hair at irradiation site. (DOCX 1533 kb) [file 13014_2017_864_MOESM3_ESM.docx]

**Additional file 3: Figure S2** Picture of a BB-treated mouse head 6 months post-irradiation. The arrow points to the circle of gray hair at irradiation site.
